# Does the pharmacy expenditure of patients always correspond with their morbidity burden? Exploring new approaches in the interpretation of pharmacy expenditure

**DOI:** 10.1186/1471-2458-10-244

**Published:** 2010-05-11

**Authors:** Amaia Calderón-Larrañaga, Beatriz Poblador-Plou, Anselmo López-Cabañas, José Tomás Alcalá-Nalvaiz, José María Abad-Díez, Daniel Bordonaba-Bosque, Alexandra Prados-Torres

**Affiliations:** 1 Aragon Health Sciences Institute (I + CS), Zaragoza, Spain; 2 Department of Statistical Methods, Science Faculty, University of Zaragoza, Zaragoza, Spain; 3 Department of Health and Consumer Affairs, D.G. Planning and Assurance, Government of Aragon, Zaragoza, Spain

## Abstract

**Background:**

The computerisation of primary health care (PHC) records offers the opportunity to focus on pharmacy expenditure from the perspective of the morbidity of individuals. The objective of the present study was to analyse the behaviour of pharmacy expenditure within different morbidity groups. We paid special attention to the identification of individuals who had higher values of pharmacy expenditure than their morbidity would otherwise suggest (i.e. outliers).

**Methods:**

Observational study consisting of 75,574 patients seen at PHC centres in Zaragoza, Spain, at least once in 2005. Demographic and disease variables were analysed (ACG^® ^8.1), together with a response variable that we termed 'total pharmacy expenditure per patient'. Outlier patients were identified based on boxplot methods, adjusted boxplot for asymmetric distributions, and by analysing standardised residuals of tobit regression models.

**Results:**

The pharmacy expenditure of up to 7% of attendees in the studied PHC centres during one year exceeded expectations given their morbidity burden. This group of patients was responsible for up to 24% of the total annual pharmacy expenditure. There was a significantly higher number of outlier patients within the low-morbidity band which matched up with the higher variation coefficient observed in this group (3.2 vs. 2.0 and 1.3 in the moderate- and high-morbidity bands, respectively).

**Conclusions:**

With appropriate validation, the methodologies of the present study could be incorporated in the routine monitoring of the prescribing profile of general practitioners. This could not only enable evaluation of their performance, but also target groups of outlier patients and foster analyses of the causes of unusually high pharmacy expenditures among them. This interpretation of pharmacy expenditure gives new clues for the efficiency in utilisation of healthcare resources, and could be complementary to management interventions focused on individuals with a high morbidity burden.

## Background

Analyses of trends in pharmacy expenditures are frequently carried out from a population perspective, focusing on the gross increase in costs [1,2]. However, the computerisation of primary health care (PHC) records and the resulting large databases offer an excellent opportunity to address this problem considering the health characteristics of individual patients [3] such as the type and burden of morbidity [4].

The development of patient classification systems has enabled measurement and profiling the morbidity of patients and populations according to their complexity and expected utilisation of healthcare resources [5]. That is, patients with similar morbidity profiles are likely to require equal delivery of healthcare resources (e.g. prescriptions). The Adjusted Clinical Groups (ACGs) system developed at Johns Hopkins University (Baltimore, MD, USA) is considered to offer great potential within PHC due to its holistic and longitudinal approach in characterising the morbidity burden of patients as well as its capacity to describe the case-mix of a reference population [6,7]. The commonest applications of ACGs comprise assessment of provider performance, allocation and utilisation of resources, and outcomes analyses [8,9].

When analysing pharmacy expenditure, the tools mentioned above can also be used to study the behaviour of expenditure within the different categories of health status. Further, focusing on individuals whose expenditure is higher than the average within one specific category of health status seems to be of particular interest. This group of individuals, statistically named as 'outliers' [10], can also be understood as those whose pharmacy expenditures exceed their burden of morbidity. This new approach is important: (i) for identifying patients for whom pharmacy care and its potential cost may be optimised; and (ii) because it opens up new research channels related to the characterization and prediction of patients and professionals on whom cost-control measures should be focused.

The objective of the present study was to analyse the behaviour of pharmacy expenditure within different morbidity groups. We paid special attention to the identification of individuals who had higher expenditure values than their morbidity would otherwise suggest.

## Methods

A retrospective observational study was conducted based on information provided by computerised clinical records from six PHC centres in Zaragoza, Spain, in 2005. In an effort to obtain a homogeneous sample with respect to the quality of the records, we only selected centers with ≥ 2 years of experience in the use of computerised clinical records (as compiled through the OMI-AP system). We only included patients aged > 14 years who had been assigned to these centres and who attended such centres at least once during the study year. The final study population was composed of 75,574 patients.

Data were obtained from administrative registries of the Aragon Health Care System (Zaragoza, Spain) after official request and authorization. Personal information was made anonymous according to the Spanish Organic Law of Personal Data Protection 15/1999. This work was part of a project funded by the Carlos III Health Institute, which was approved by the Ethics Committee for Clinical Investigation of Aragon (CEICA).

### Study variables

Demographic variables (age, sex), pharmacy expenditure, and burden of morbidity were recorded for each patient. For the latter, all diagnoses or reasons for visits assigned to each patient during the study period were coded according to the International PHC Classification (IPHCC) [11]. Subsequent conversion ('mapping') was made from the IPHCC to the International Disease Classification (ICD-9-CM) [12].

Based on the variables of age, sex and diagnosis registered along the study year, a single ACG category was assigned to each patient [13]. The ACG system (version 8.1^®^) assigns all ICD-9-CM codes to one of 34 diagnosis clusters known as 'Aggregated Diagnosis Codes' (ADGs). Individual diseases or conditions are placed into a single ADG cluster based on the: duration of the condition (e.g. acute, recurrent, or chronic); severity of the condition (e.g. minor and stable versus major and unstable); diagnostic certainty (symptoms versus documented disease), aetiology of the condition (infectious, injury, or other); and specialty care involvement (e.g. medical, surgical, obstetric, haematologic). Each ADG is a grouping of diagnosis codes that are similar in terms of severity and likelihood of persistence of the health condition treated over a relevant period of time. In a second stage, the ACG methodology uses a branching algorithm to place subjects into one of 106 discrete categories based on their assigned ADGs, their age, and their sex. The result is that individuals within a given ACG have experienced a similar pattern of morbidity and resource utilisation over a given year. For the sake of parsimony, ACGs with similar expected use of resources are aggregated into 'morbidity bands' (i.e. low, moderate or high).

The primary measurement variable, which we termed 'total pharmacy expenditure per patient', included the retail price of medicines, supplies and accessories consumed by patients. This was obtained by combining data between the billing database of the pharmacy offices and the OMI-AP patient database. The tables were cross-referenced via the health card identifier (a sequential numeric tag assigned to each patient). All identifying data were removed to guarantee confidentiality.

### Identification of outlier patients

There are many statistical procedures for identifying outlier data. Most are designed to prevent a 'masking' effect by groups of outliers or to be applied with multivariate data [14]. The aim of the present study was to identify subgroups of 'unusual' patients in terms of their pharmacy expenditure considering their morbidity burden given the absence of a well-defined external cutoff, rather than the deletion of suspicious data or a robust estimation of location or scale parameters. The applied methods were chosen on the basis of a balanced compromise between their simplicity and their adequacy to the nature of the dependent variable 'pharmacy expenditure'. The statistical procedures for identifying outlier data are detailed below.

#### (i) Stratified boxplot by ACG (BXP)

One of the most frequently used graphical techniques for visualising the distribution of continuous univariate data is the boxplot, which was originally proposed by Tukey [15]. This tool gives information about the location, spread, skewness and tails of data. This last application consists of detecting the atypical cases of a distribution based on the following formula:

where *Q*_3*i *_and *Q*_1*i *_are quartiles three and one, respectively, of the distribution of pharmacy expenditure for each ACG. Patients whose spending during the study period exceeded the trimming point (*T_i_*) respective to their ACG category were considered to be outliers.

The constant 1.5 is the commonest value applied in Boxplot techniques. It is widely acknowledged that 0.35% of normally distributed data exceeds the trimming point (*T_i_*) for moderate sample sizes (rate of false-positives). This percentage can be highly increased in non-normal or asymmetric distributions.

#### (ii) Adjusted boxplot for asymmetric distributions and stratified by ACG (Adj. BXP)

For asymmetric distributions (as is the case for pharmacy expenditures), the BXP method tends to identify too many extreme cases (outliers) because the trimming points are derived from a normal distribution [16]. In these cases, Hubert et al. [17] proposed a generalisation of the boxplot that includes a robust asymmetry measurement to determine the trimming limits or points, which they called function *h_r _(Medcouple, MC)*. The medcouple is defined as:

where *x*_1 _and *x*_2 _are independently sampled cases of the asymmetric distribution *F, m*_*F *_is the median of *F*, and *h *is the kernel function given by the following formula:

Thus, the upper limit of the intervals is given by:

According to this same study, the family of exponential models should be chosen to generalise function *h_r _(MC).*

#### (iii) Standardised residuals (RESID)

Another method for detecting individuals with atypical values in the dependent variable (Y) and which is widely used for regression model diagnostics is the residual analysis:

where *y*_i _are the observed values and  the predicted values of Y.

Standardization of these residuals is applied to eliminate the effect of the measurement units on account of the dependent variable [18]. If the normality assumption is fulfilled, the percentage of standardised residuals that fall out of the interval ± 2 is ~5%. Thus, cases with a residual > 2 are considered 'extreme' and therefore identified as outliers [19].

Regarding the regression model, the logarithm of pharmacy expenditure was used as the dependent variable to standardise the distribution [20]. The independent 'ACG category' variable was introduced into the model as a group of dummy variables. The 'pharmacy expenditure' interest variable had a truncation point in the nil expenditure (7.8% of the population recorded nil expenditure), so truncated tobit models were applied [21]. In these cases, the distribution of the variable in question is modelled as a mixture of a continuous distribution and a discrete distribution [22].

### Demographic, clinical, and pharmacy-expenditure characteristics of outlier patients

A descriptive analysis was first carried out on a global basis and for each group of patients (normal/outliers) whereby the mean, median, and variation coefficient of the continuous variables and the distribution of frequencies of the categorical variables were calculated. The differences between outlier patients and the rest were analysed as well as the differences between outlier groups themselves (dependent upon the applied identification method) via the Mann-Whitney U test if the variable was continuous but did not follow a normal distribution (age, mean annual expenditure per patient, and mean annual number of visits per patient) and by a chi-square test for the qualitative sex and morbidity burden variables. Later, a residual study for these latter variables was undertaken in the event of significant differences.

Given the large sample size, interpreting differences in terms of statistical significance would lead to misinterpretations. Evaluation of the relevance and magnitude of the differences between normal and outlier patients and among outlier groups themselves was carried out based on 'comparison indices' (Table 1). Calculation of these indices required an indirect adjustment using the general population as a reference. Based on this information, we calculated three ratios. First, we calculated the 'observed/average' ratio. This divides the annual average expenditure per patient in each specific group by the annual average expenditure per patient within the general population. This indicates the extent to which a certain group of patients has a higher expenditure relative to the general mean. Second, we calculated the 'expected/average' ratio. Bearing in mind the burden of morbidity, this ratio divides the expected expenditure for each specific group by the expenditure of the general population. From this we deduce the degree to which a certain subgroup of the population exhibits a higher or lower burden of morbidity than the mean of the general population. Third, we calculated the 'observed/expected' ratio. This ratio divides the annual average expenditure per patient observed in each specific group by the annual average expenditure per patient expected in those same groups given the burden of morbidity. This indicates the mean expenditure of each subgroup of patients compared with the mean expenditure of the general population, adjusting for morbidity.

**Table 1:** Demographic, clinical, and pharmacy-expenditure characteristics of outlier patients according to the identification method (n = 75,574).

	General population	Patients with normal pharmacy expenditure			Outliers		
		
		BXP	Adj. BXP	RESID	BXP	Adj. BXP	RESID
**Demographic characteristics**							

Average age	49.3	49.2^a^	49.3^a^	49.3^a^	50.3^abc^	48.01^ab^	47.5^ac^
Women (%)	55.8	55.8	55.8	55.9^a^	56.3^bc^	56.5^bd^	51.0^acd^
Patient proportion (%)	100	93.5	98.3	98.8	6.5	1.7	1.2

**Pharmacy expenditure characteristics**							

Annual average expenditure/patient (€)	413.1	334.7^a^	376.4^a^	388.19^a^	1,548.2^abc^	2,509.2^abd^	2,434.3^acd^
Proportion of total expenditure (%)	100	75.8	89.6	92.8	24.2	10.4	7.2

**Morbidity bands**							

Low (%)	22.3	21.7^a^	22.0^a^	22.0^a^	**30.5**^**abc**^	**34.7**^**abd**^	**39.2**^**acd**^
Moderate (%)	57.9	57.7^a^	**58.0**^**a**^	57.9^a^	**59.6**^**abc**^	53.0^abd^	55.6^acd^
High (%)	19.9	**20.6**^**a**^	**20.0**^**a**^	**20.1**^**a**^	9.9^abc^	12.3^abd^	5.2^acd^

**Comparison indices**							

Observed/Average	1	0.8	0.9	0.9	3.8	6.1	5.9
Expected/Average	1	1.1	1.1	1.1	0.7	0.6	0.4
Observed/Expected	1	0.7	0.8	0.9	5.4	10.1	13.3

The *p*-value corresponds to the chi-square test in the case of percentages or to the Mann-Whitney U test for continuous variables:

^a ^Significant differences between normal and outlier patients (p < 0.05)

^b ^Significant differences between outlier patients identified by BXP and Adj. BXP (p < 0.05)

^c ^Significant differences between outlier patients identified by BXP and RESID (p < 0.05)

^d ^Significant differences between outlier patients identified by Adj.BXP and RESID (p < 0.05)

The associations between the morbidity bands and the type of patient were calculated by analysing standardised residuals of the chi-square test:

**BOLD TEXT **Significant association between categories (p < 0.05)

The STATA 10 software package was used to calculate the truncated tobit models, R free software to calculate function *h_r _(Medcouple, MC) *and SPSS 15.0 for the remainder of our statistical analyses.

## Results

The general population (n = 75,574) was composed of 55.8% women with an average age of 49.3 years (confidence interval (CI) 95% 49.1-49.4). More than one-quarter of the population was aged > 64 years, and 66.9% of the assigned population of the centre attended during the study year. The population was grouped in 67 ACGs consisting of an average of 1128 individuals. The standard deviation of pharmacy expenditure within ACGs was very heterogeneous, ranging from 58.3€ up to 2919.0€. The variation coefficients by ACGs ranged from 0.9 to 6.6 (mean, 2.6). The variability observed within the morbidity bands is summarised in Table 2.

**Table 2:** Summary of the variation in pharmacy expenditure within morbidity bands.

Morbidity bands	N° of patients	Mean (€)	Std. Dev. (€)	VC	**Min**-**Max (€)**	Median (€)	**Q**_**1**_-**Q**_**3 **_**(€)**
Low	16,818	75.1	238.61	3.2	0.0-9,110.8	15.4	3.5-45.7
Moderate	43,453	383.7	779.14	2.0	0.0-35,993.1	109.0	25.3-469.8
High	15,302	877.2	1,132.76	1.3	0.0-35,113.4	549.1	135.5-1,230.0

VC: variation coefficient

Q1: first quartile

Q3: third quartile

### Comparisons between normal patients and outlier patients with regard to the annual pharmacy expenditure

There were statistically significant differences between normal patients and outlier patients (regardless of the method used to identify these groups). This is corroborated by the observed/average ratios which, for this group, suggested pharmacy expenditures 3.8-6.1-times greater than the global mean (Table 1, observed/average ratios). The analysis of the standardised residuals of the chi-square test indicated that there was a significantly higher presence of outlier patients within the low-morbidity band which matched up with the higher variation coefficient observed in this group (3.2 vs. 2.0 and 1.3 in the moderate- and high-morbidity bands, respectively). The expected/average ratio showed that the outlier population was 30-60% less ill than the general reference population (Table 1, expected/average ratios). As a result, the observed/expected ratios were 5-13-times greater for outlier patients than for patients with a normal expenditure (Table 1, observed/expected ratios).

### Comparisons between groups of outlier patients dependent upon the identification method

**Figure 1:**
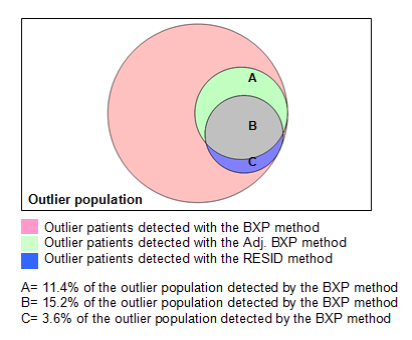
**Overlapping subsets among outlier populations as detected by three statistical methods**.

**Figure 2:**
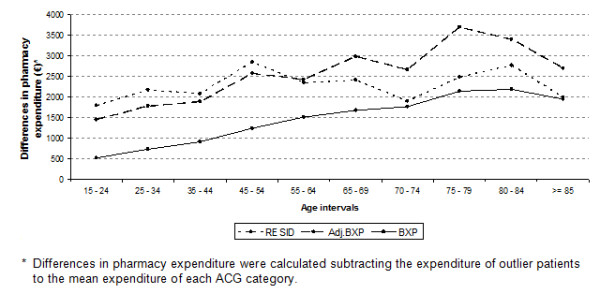
**Differences in pharmacy expenditure of outlier patients relative to the mean expenditure expected for each ACG category, by age groups**.

The first noticeable finding was the difference in the number of patients detected by each method. Whereas the BXP method detected 6.5% of the population, the Adj. BXP and RESID methods detected 1.7% and 1.2%, respectively (Table 1). Second, these last two methods detected subpopulations of the group that was detected by the BXP method (Figure 1). Third, patients detected by the Adj. BXP and RESID methods had expenditures 800-1000€ higher per year than patients detected by the BXP method. These differences were statistically significant and became evident in the observed/average ratios (Table 1, observed/average ratios). Figure 2 emphasizes these differences, also illustrating increasing differences by age. A detailed analysis of the different burdens of morbidity among these three groups of patients reveals that patients detected by the Adj. BXP and RESID methods were 10-30% less sick than the population identified by the BXP method (Table 1, expected/average ratios). Fourth, differences were noticed in the 'proportion of total expenditure/patient proportion' ratios for the BXP method and the Adj. BXP and RESID methods (ratios 3.7; 6.1 and 6.0, respectively). The ratios between the observed and expected expenditure as detected by the Adj. BXP and RESID methods were almost twofold greater than that for the BXP method (Table 1, observed/expected ratios).

## Discussion

The results of the present study indicated that pharmacy expenditure of up to 7% of the population attending the studied PHC centres during one year exceeded expectations given their morbidity burden, and that this group of patients was responsible for up to 24% of the total annual pharmacy expenditure. This information may be of interest in the context of growing concern about the increase in pharmacy expenditures in PHCs, and the application of possible measures for efficient and rational management of drug prescriptions.

Among the existing pharmacy cost-containment policies in Europe, some are focused on one of the key stakeholders in healthcare: the prescriber. This is achieved through the analysis of the quality and costs of their prescribing [23]. Several countries disseminate reviews of drug utilisation to general practitioners (GPs) on the basis that prompt, detailed feedback of individualised prescribing data may be effective in rationalising the use of drugs [24]. The concepts and methodologies shown in the present study could be incorporated in the routine monitoring of the prescribing profile of GPs. This could be used to evaluate not only their performance, but also to target groups of outlier patients and foster analyses of the causes of unusual pharmacy expenditures among them, thereby furthering the design of specific interventions.

The results of the present study indicate that there was a potential margin to improve drug utilisation in the group of patients with a low morbidity burden. That is, there was a lower correspondence between the morbidity profile of patients and their expenditure in those groups of patients who showed a low morbidity burden. A wider variation of pharmacy expenditure was observed within the group of patients with a low morbidity burden (variation coefficient: 3.2 vs. 2.0 and 1.3 in the moderate- and high-morbidity bands, respectively), leading to a higher number of patients whose figures were far removed from the expenditure of most patients belonging to the same group (i.e. outliers). The expenditure of this group of outliers was up to 13-times higher than expected given their burden of morbidity. This interpretation of pharmacy expenditure gives new clues for the efficiency in utilisation of healthcare resources, and could be complementary to management interventions focused on individuals with a high morbidity burden [25,26].

The trimming methods of the present study have been widely applied in the hospital setting to avoid incorporating outlier cases when valuating the mean utilisation of resources of a given healthcare resource group [27,28]. However, analysing the group of outliers itself deserves special attention. All three methods are easy to implement, adjusted by morbidity burden and adapted to the large sample size and the non-parametric distribution of the studied variable pharmacy expenditure, which is why other tests (e.g. Grubbs' test, Dixon's Q test) were not used. The three methodologies identify subgroups of the same population, reinforcing the robustness of the selected techniques. However, which method best identifies the group of outlier patients? The empirical evidence suggests that the most sophisticated statistical methods, such as the Adj. BXP for asymmetric distributions or standardised residuals, offer more specificity in identifying outlier patients. The average expenditure of patients detected by these two methods was 800-1000€ higher per year than that of patients detected by the classic BXP method. Moreover, these expenditures had a lower correspondence with their burden of morbidity as reflected in the ratios between the observed and expected expenditure, which were almost twofold greater than that given by the classic BXP method.

According to the World Health Organisation, the rational use of drugs requires that patients receive medications appropriate to their clinical needs at the lowest cost to them and their community. The evidence indicates that there may be factors associated to the practitioner and the patient which deviate them from the rational use of drugs, resulting in an unjustifiable use of scarce resources and widespread health hazards. Among the variables related to the practitioner, the continuous training, the influence of the pharmaceutical industry, and the 'burnout syndrome' as a consequence of chronic stress at the workplace have been identified most frequently [29]. Regarding patient-related factors, the pressure that they can occasionally exert to obtain medications that 'cure' their health problem has been discussed [30,31]. This problem is aggravated when practitioners' time to see a patient is limited [32]. The pressure can be very difficult to handle because, in most cases, there is little time to explain the preferred treatment option of GPs to sceptical patients [33].

These findings must be interpreted with caution due to several reasons that may affect their internal and external validity. The main potential limiting factor of the present study could be related to the quality of the diagnostic information registered in the electronic medical records. Even if a series of inclusion criteria were applied during the health centre selection process to guarantee the quality and reliability of clinical data, a two-year period of experience in the use of electronic records may be insufficient. This could explain why patients have pharmacy expenditures for diseases that are not registered in the medical records of GPs, thus over-estimating the lack of correspondence between expenditure and morbidity burden of the population. Using more recent data is required to confirm the internal validity of the results.

Second, there were limitations derived from the study design owing to the restricted access to information. That is, drug use was evaluated only in terms of the expenditure. This ignored aspects related to the number of drug prescriptions or the number of defined daily doses. This variable would have been of great help when analysing the causes of excess use of drugs within the same morbidity group.

A third factor that could compromise the internal validity of the results obtained in the present study was related to the explanatory capacity of the tool used to measure morbidity: ACGs. It could be argued that the ACGs perform weakly in low-morbidity bands or, further, that updates on the effectiveness and cost-effectiveness of health technologies (e.g. drugs) were not considered in the design process. Nevertheless, the statistical power of the ACG system developed at Johns Hopkins University has been internationally validated [34-37] and its different versions are regularly updated [13].

Fourth, the results of the present study correspond to a population treated in urban health centres. They are therefore not fully representative of the population treated across an entire health system at the PHC level, thus affecting their external validity. Nevertheless, the characteristics of the studied sample, such as the average number of diagnoses per patient [38], the intensity of use [5,39], the proportion of women, the aging rate, and the distribution of patients according to ACGs [40] fit in with those reported in national and international studies.

If the methodology of the present study is to be used to target patients and prescribers amenable to intervention strategies, they should first be validated. This implies a clinical validation by expert panels as well as carrying out economic studies that shed light on the potential savings of this type of interventions.

## Conclusions

We began this contribution by asking if the pharmacy expenditure of patients always corresponds with their morbidity burden. The present study identified a group of patients with low morbidity whose pharmacy expenditure exceeded expectations. Identification of the factors that influence this excess in pharmacy expenditure can improve the adaptation of pharmacy services and increase the efficiency of the PHC system.

## Competing interests

The authors declare that they have no competing interests.

## Authors' contributions

ACL, ALC and APT generated the research question. BPP, JTAN and DBB carried out the statistical analyses. ACL, BBP, ALC, JMAD and APT participated in the interpretation and discussion of results. ACL, ALC and APT contributed to the drafting of the manuscript. ACL coordinated writing of the contribution. All authors read and approved the final manuscript.

## Pre-publication history

The pre-publication history for this paper can be accessed here:

http://www.biomedcentral.com/1471-2458/10/244/prepub
